# A Novel Faster-Acting, Dry Powder-Based, Naloxone Intranasal Formulation for Opioid Overdose

**DOI:** 10.1007/s11095-022-03247-5

**Published:** 2022-04-06

**Authors:** Tair Lapidot, Mohammed Bouhajib, Janice Faulknor, Shabaz Khan, Galia Temtsin Krayz, Carolina Abrutzky, Dalia Megiddo

**Affiliations:** 1 Nasus Pharma, Harakevet 29, 6618306 Tel Aviv, Israel; 2 Pharma Medica Research Inc., 6100 Belgrave Rd, Mississauga, ON L5R 0B7 Canada; 3 Formerly of Pharma Medica Research Inc., 6100 Belgrave Rd, ON L5R 0B7 Mississauga, Canada; 4 Formulex Pharma Innovation Ltd, 18 Einstein Street, 7403622 Nes Ziona, Israel

**Keywords:** bioavailability, dry powder, naloxone microspheres, nasal spray, lactose

## Abstract

**Objective:**

To examine the pharmacokinetics and safety of FMXIN001, a new intranasal powder-based naloxone formulation, in comparison to Narcan® nasal liquid spray.

**Methods:**

FMXIN001, was developed by blending drug microspheres with larger lactose monohydrate particles, that serve as diluent and carrier, as well as a disaggregating agent. Scanning electron microscopy and X-ray were used to characterize the formulation and *in vitro* deposition was investigated using a nasal cast.

We compared the pharmacokinetics and safety of FMXIN001 versus Narcan® in two clinical trials: a pilot study with 14 healthy adults and a pivotal trial in 42 healthy adults (NCT04713709). The studies were open-label, single-dose, randomized, two-period, two-treatment, two-sequence crossover studies to assess the pharmacokinetics and safety of FMXIN001 versus Narcan® nasal spray.

**Results:**

FMXIN001 comprises naloxone microspheres (5-30 μM) and lactose particles (40–240 μM). Upon *in vitro* testing, naloxone deposits mainly to the middle turbinates region and the upper part of the nasal cavity of a nasal cast. In human subjects, FMXIN001 produced significantly higher exposure at the initial time points of 4, 10, and 30 min, post-administration, compared to Narcan®. Both treatments were safe and well tolerated. FMXIN001, powder-based spray, results in similar overall exposure to Narcan®, but with more rapid absorption in the first 30 min.

**Conclusions:**

FMXIN001 is expected to have a shorter onset of action for a more effective therapeutic intervention to manage opioid overdose. Rapid administration of naloxone in cases of opioid overdose is imperative, given the alarming increase in mortality rates.

**Supplementary Information:**

The online version contains supplementary material available at 10.1007/s11095-022-03247-5.

## Introduction

The surface area of the nasal mucosa in humans is around 150 cm^2^, a tissue which is well supplied by blood vessels. This ensures a rapid absorption of many drugs generating high systemic blood levels whilst avoiding first pass metabolism. An important attribute of intranasal administration is that it is non-invasive and easy for care-givers administer, or to self-administer. There is also lower potential for injuries or disease transmission (hepatitis B, HIV) compared to an injectable product. This is of special importance if fast relief from severe symptoms is required and patient’s ability to deal with injections is impaired.

Nasal powder formulations are generally simple compositions with or without excipients, which allow for the administration of larger drug doses and enhance drug diffusion and absorption across the mucosa, improving bioavailability compared to nasal liquids [1]. Addition of lactose excipient, for example, to the formulation increases the fluid volume on the nasal mucosal surface by increasing the osmotic pressure, which can improve absorption of some drugs [2]. Excipients may also confer better stability and longer shelf-life as compared to the fluid nasal sprays.

Misuse and abuse of opioids has evolved into a worldwide epidemic, particularly in the United States (US) where it was declared a national public health emergency in October 2017 [3–6]. Rapid and effective administration of naloxone in cases of opioid overdose is imperative, based on the growing use of potent synthetic opioids and alarming increase in mortality rates.

Naloxone hydrochloride is the opioid antagonist most commonly used for the complete or partial reversal of opioid overdose, including respiratory depression, sedation, and hypotension. It was approved by the Food and Drug Administration (FDA) in 1971, and later also by other regulatory agencies, for intravenous (i.v.), intramuscular (i.m.), and subcutaneous (s.c.) administration. It is a pure opioid antagonist with competitive action and extremely high affinity to the μ-opioid receptors in the central nervous system (CNS). In the absence of narcotics or agonistic effects of other narcotic antagonists, it exhibits essentially no pharmacologic activity and has no abuse potential [7, 8].

Intranasal (IN) administration is quick, non-invasive and easy to use, as compared to parenteral injection, and protects against accidental intra-vessel injection. Intranasal naloxone was approved by the FDA in 2015: Narcan® Nasal Spray (Adapt Pharma, PA, USA) at a dose of 4 mg in 0.1 mL per one spray. In 2017, Nyxoid® 1.8 mg Nasal Spray (Mundipharma Corporation, Ireland) was authorized for marketing in the European Union. The aforementioned liquid-based delivery systems, however, suffer from variable absorption owing to a large fraction of the delivered drug being deposited in the lower anterior segment of the nasal cavity, in front of the nasal valve [9]. This anterior segment is predominantly lined with non-ciliated squamous epithelium that is less permeable to drugs than the respiratory mucosa beyond the nasal valve [10].

It has been reported that powder-based intranasal formulations may reach the blood stream faster and have better bioavailability than liquid sprays due to significantly larger deposition in the nasal mucosa [2, 9, 11]. Faster naloxone absorption is paramount when treating opioid overdose since this is an emergency situation with victims suffering respiratory depression for an unknown period, and at a high risk of death if not treated promptly.

In addition, recent studies of intranasal naloxone showed that about a third of the treated victims of opioid overdose will need at least 2 doses of naloxone (4 mg) and about 16% will need a third dose [12]. Moreover, the increased use of illicit new synthetic potent opioids may necessitate higher doses of naloxone and quicker onset of action. [8, 13].

Spray drying is an excellent method for the production of dry powders for inhalations since particle size distribution and residual moisture content of the spray dried powders can be easily controlled by the process conditions. In addition, the processing of heat sensitive pharmaceutics is feasible owing to the cooling effect of solvent evaporation [14]. However, the agglomeration of spray dried particles is common due to the high atomization energy and particle collisions. In the current study, therefore, lactose monohydrate excipient is used to prevent the aggregation. This excipient, approved for oral inhalation products, was blended in-situ with dried Naloxone microspheres to provide a novel stable nasal formulation. The formulation comprises two populations, small Naloxone hydrochloride particles and large Lactose monohydrate particles, evident in the formulation’s particle size distribution.

This study demonstrated the preparation and characterization of FMXIN001 a new dry powder-based delivery system and aimed to prove its bioavailability in comparison to Narcan® Nasal Spray, as well as demonstrate its advantage for treatment of opioid overdose patients.

## Methods

### FMXIN001 Naloxone Powder Preparation

Lactose monohydrate was obtained from Meggle Pharma; naloxone hydrochloride dihydrate (from Noramco); ethanol (from BioLab).

The manufacture of FMXIN001 is based on a modified spray drying process using a Mini Spray Dryer B-290 (Büchi Labortechnik AG.). Briefly, Naloxone hydrochloride dihydrate (3.0 g) was dissolved in an ethanol-water mixture (50:50) with stirring. The clear and homogeneous solution of naloxone hydrochloride is spray-dried using the Mini Spray-Dryer and mixed with lactose monohydrate to yield a product with a final homogeneous level of 20% w/w naloxone hydrochloride dihydrate. The final composition is then introduced into a disposable unit dose device, Aptar Pharma (France), to provide a precise dose of 4 mg naloxone hydrochloride dihydrate upon single intranasal administration.

FMXIN001 naloxone powder was manufactured and loaded into the Aptar Unit dose powder devices, by Formulex Pharma Innovations (Israel).

### Optimization of Excipient Amount

The administration of nasal powder formulations has been associated with greater sensory irritation than liquid sprays and the amount of powder is recommended to be kept as low as possible, preferably about 20 mg or below [15].

The Aptar Unit Dose Powder Device has a maximum fill volume of 130 mm^3^ allowing to load a fill weight of 10–80 mg of the powder. A fill weight of 20 mg was selected, therefore, to prevent possible aggregation and to keep the sensory irritation as low as possible. Lactose monohydrate is the only excipient in the formulation and its amount was established as 16 mg in the device.

### Formulation Analysis - HPLC

A stability indicating HPLC method was developed and validated for naloxone HCl assay analyses.

The method employed a Waters ACQUITY H-class HPLC system with UV photodiode array detector (PDA). The output signal was processed using Waters Empower 3 software. A Phenomenex Gemini C18 4.6 × 250 mm column with 5 μm particle size was used. The separation was achieved using an isocratic method. Mobile phase was prepared by dissolving of 1.36 g of sodium 1-octanesulfonate in 580 mL of water and mixing with 420 mL of methanol and 1.0 mL of phosphoric acid. The flow rate of the mobile phase was 1 mL/min. Naloxone HCl sample and standard were dissolved in diluent prepared from dissolution of 75 mg of edetate disodium and 0.45 mL hydrochloric acid in 1 L of water. The run time was 15 min.

The related substances were analyzed by the dedicated method, which was also validated. The same HPLC system was used. An ACQUITY UPLC CSH C18 2.1 × 150 mm column with 1.7-μm particle size was applied. The solution A was prepared from Sodium 1-octanesulfonate buffer pH 2.0, Mobile phase A and B were prepared from Solution A/THF/ACN at different ratios. The gradient program (min/%B) was set as 0/9%, 13/ 30%, 13.1/0%, 24/100%, 24.1/9% and 27/9%. The injection volume was 2.0 μL. The column temperature was set at 35°C and the PDA detection was at 229 nm.

The method is also capable of separating and quantifying the naloxone-related compounds listed in the USP monograph of naloxone HCl in nasal spray formulations.

Both methods were validated per USP <1225> and deemed suitable for intended use per the results shown in the on-line resource Table S.I.

### Formulation Analysis - Scanning Electron Microscope

A FEI Quanta-200 Scanning Electron Microscope (SEM) equipped with an Everhart-Thornley Detector was used to obtain the images of the spray-dried powder. The accelerating voltage of 20 kV was applied to provide magnification from 250 to 10,000 times. In addition, an X-ray Element Analysis Detector (Link ISIS, Oxford Instruments, GB) was used to determine the drug and particle identity and their distribution. Particle size was measured using the Malvern Mastersizer 3000 series based on the Light Diffraction method.

### Formulation Analysis – X-Ray

Phase analysis was by X-ray powder diffraction (XRPD). The data were collected on a Panalytical Empyrean powder diffractometer (Cu Kα radiation, λ = 1.54178 Ǻ) equipped with an X’Celerator linear detector and operated at V = 40 kV, I = 30 mA. Scans were run in a 2q range of 3–38° with step equal to ~0.0167°, scan speed ~0.042°/sec. Peak lists were automatically generated using Match! 2 p-XRD analysis software.

### *In Vitro* Nasal Cast Deposition

The aim of the nasal cast studies was to explore the relative distribution of powder in the different nasal cavity regions and the potential for powder particles to reach beyond the nasal cavity into the nasopharynx; airways and lungs [16, 17]. Three trials were performed for naloxone (4 mg in 20 mg of powder) delivered by a Unit Dose powder device (UDSp- APTAR) using a 45° orientation and an insertion of 15 mm into the nostril of a Caucasian male nasal cast. The nasal cast is divided into a series of four blocks and a filter which mimics the nasal anatomy and enables accurate measurement of drug deposition in each region. A specific “jig” which assured the holding angle of 45°; the angle from the center wall of 4° and an insertion depth of 15 mm was utilized. One dose was delivered into each nostril using UDSp. Samples were collected from the nasal cast by rinsing each region of interest with a defined volume of water. All the samples were analyzed by using spectrophotometer set at 229 nm absorbance and a calibration curve generated with naloxone microsphere powder standard solutions. Shot weight was recorded to check the proper actuation of all the devices.

### Clinical Studies - Aim

A pilot study and a pivotal clinical study were performed in 14 and 42 healthy volunteers, respectively, according to a similar protocol. The primary objective in both studies was to evaluate the comparative bioavailability of naloxone between FMXIN001 Naloxone Microspheres Powder 4 mg for Nasal Application (Nasus Pharma, Israel) and Narcan® (naloxone HCl) NASAL SPRAY 4 mg (Adapt Pharma, Inc., USA) after a single-dose in healthy subjects under fasted conditions. The secondary objective was to evaluate the safety and tolerability of the study treatments (NCT04713709). The pilot study results (see on-line resource Fig. S3 and Table S.II) supported progression to the pivotal study, described here.

Both studies were performed in accordance with current International Council for Harmonisation (ICH) Good Clinical Practice (GCP). Written consent was obtained from each subject before entering the study.

### Pivotal Clinical Study Design & Setting

This was an open-label, single-dose, randomized two-period, two-treatment, two-sequence, crossover study, designed to evaluate the comparative bioavailability of naloxone between a test and reference product in healthy subjects under fasted conditions. In each period, following an overnight fast, subjects were randomly administered a single intranasal dose (one actuation) of the test or reference product.

Concentrations of unconjugated naloxone were measured by LC MS/MS from plasma samples collected over an 8-h interval after dosing in each period. The PK variables C_max_, AUC_0-8h_, AUC_inf_, AUC_0-4min_, AUC_0-10min_, AUC_10-30min_, T_max_, K_el_, and T_half_ were calculated using a noncompartmental approach.

An assessment of safety (section 3.4) was based primarily on the incidence, frequency, and severity of adverse events (AEs). A validated smell test was also used to evaluate safety of the studied products.

#### Description of Materials

Treatment A: Naloxone Intranasal Spray 4 mg (FMXIN001 4 mg microspheres powder, nasal spray, containing 16 mg lactose as an excipient) (Nasus Pharma, Israel). Intranasal administration was using the Unit Dose Powder Device (Aptar Pharma, France) for one actuation in the right nostril.

Treatment B: Naloxone HCl, (Narcan® NASAL SPRAY) 4 mg (Adapt Pharma, Inc., USA) for one actuation in the right nostril.

Dosing was verified by weighing each device before and after drug administration.

#### Setting

The study was performed at the Pharma Medical Research clinical facility in Toronto, Canada. Subjects were dosed on January 31, 2021 (period 1) and February 07, 2021 (period 2). Study drugs were administered by qualified clinical staff. Subjects were randomly assigned to one treatment sequence, according to a predetermined computer-generated randomization scheme. The study was implemented by Pharma Medical Research Inc., a Canadian contract research organization.

#### Participant Characteristics

The study population included nonsmoking male and female healthy volunteers, 18 years of age or older. A summary of subject demographics is shown in Table I. Three subjects were excluded from the PK data set due to technical issues, 1 due to a COVID19 positive result.

**Table I:** Summary of Demographic Characteristics

Demographic Characteristic	Statistics	Safety Dataset N = 46	Pharmacokinetic and Statistical Datasets N = 42
Sex, n (%)	Female	25 (54.3%)	24 (57.1%)
	Male	21 (45.7%)	18 (42.9%)
Age (years)	Mean ± SD	46 ± 12	47 ± 12
	Median	48	49
	Minimum-Maximum	20–64	20–64
Age Group, n (%)	18–40	14 (30.4%)	12 (28.6%)
	41–64	32 (69.6%)	30 (71.4%)
Weight (kg)	Mean ± SD	71.7 ± 12.8	71.2 ± 12.7
	Median	70.3	70.3
	Minimum-Maximum	48.8–97.3	48.8–97.3
Height (cm)	Mean ± SD	166.9 ± 9.9	166.6 ± 10.0
	Median	166.0	165.3
	Minimum-Maximum	146.2–190.9	146.2–190.9
BMI (kg/m^2^)	Mean ± SD	25.6 ± 3.1	25.6 ± 3.1
	Median	25.5	25.5
	Minimum-Maximum	19–30	19–30
Race, n (%)	Asian	11 (23.9%)	10 (23.8%)
	Black or African American	8 (17.4%)	8 (19.0%)
	White	27 (58.7%)	24 (57.1%)
Ethnicity, n (%)	Hispanic or Latino	17 (37.0%)	15 (35.7%)
	Not Hispanic nor Latino	29 (63.0%)	27 (64.3%)

BMI, body mass index; N, number of subjects included in each dataset; n, number of subjects in respective categories; SD, standard deviation.

#### Safety Monitoring

Safety monitoring included temperature, vital signs, ECG and blood pressure pre-dose, and at 1, 2, 4 and 12 h post-dose. General health and adverse events were monitored throughout the study.

A smell test (4-item NHANES Pocket Smell Test from Sensonics International) was conducted at each check-in and at the end-of-study [18, 19].

Clinic staff, with input from the subject, completed a nasal and non-nasal questionnaire at check-in, at 1 h and 23 h post-dose. The questionnaire used a 4-point severity scale, as described, previously [20]. A nasal cavity examination was conducted at check-in and at 1 h and 23 h) post-dose by the Investigator or designate and nasal irritation scoring was completed at each nasal cavity examination. Grading criteria for the presence of nasal irritation (mucosal edema, erythema, epistaxis), ulceration, and septal perforation were as recommended by FDA for development of allergic rhinitis products [21].

#### PK Sampling and Handling

In each period, 17 samples (10 ml) were collected by direct venipuncture or from an indwelling cannula, placed in an arm vein as follows: prior to dosing (0-h) and at 0.033, 0.067, 0.1, 0.133, 0.167, 0.25, 0.333, 0.416, 0.5, 0.75, 1, 2, 3, 4, 6, and 8 h after drug administration. The pre-dose sample was collected within 60 min prior to dosing. The samples were maintained in an ice-water bath throughout sample collection and until further processing.

Within 45 min from collection, whole blood samples were centrifuged at approximately 4°C for approximately 10 min at 3000 rpm for plasma separation. Within 45 min from centrifugation, plasma was aliquoted into 2 aliquots pending total naloxone analysis and stored at −25 ± 10°C. The bioanalysis of unconjugated naloxone in the plasma samples was performed using a validated LC-MS/MS method.

#### Bioanalytical Method

Plasma concentrations of unconjugated naloxone in subject samples were measured utilizing Analyst® Software Version 1.6.3, according to an achiral, liquid chromatographic tandem mass spectrometric detection (LC-MS/MS) method developed and validated at the Bioanalytical Laboratory of Pharma Medica Research Inc. The method involved a liquid-liquid extraction. Plasma samples (0.2 mL) were extracted under basic conditions with an organic solvent; the organic phase was dried, reconstituted and transferred for LC-MS/MS analysis. Sample analysis was conducted using reversed phase chromatography. Naloxone was analyzed in the SCIEX API 5000 mass spectrometer using positive ion scan mode. For analyte and the internal standard, the parent-daughter mass to charge ion transitions are 328–212 and 333–212 respectively. The method was successfully validated over a calibration range of 0.0100–15.0 (ng/mL). The validation included intra- day and inter-day Precision (results range 0.5–8.6%) and Accuracy (results range 86.4–102.5%), Recovery (70.6–86.1%), Specificity, Matrix effect, Selectivity with potential concomitant medication and metabolite, Dilution effect, Batch size impact, Stability during the sample processing and long period storage.

#### PK Analysis

The following PK variables were estimated using a non-compartmental approach for unconjugated naloxone: C_max_, AUC_0-8h_, AUC_inf_, AUC_0-4min_, AUC_0-10min_, AUC_10-30min_, T_max_, K_el_, and T_half._

#### Statistical Methods

Analysis of variance (ANOVA) was performed on log-transformed plasma unconjugated naloxone AUC_0-8h_, AUC_inf_, AUC_0-4min_, AUC_0-10min_, AUC_10-30min_, C_max_, and untransformed T_half_ variables. The PROC GLM procedure from SAS® (version 9.4) was used. Based on log-transformed data, ratios of the geometric means for treatments and the corresponding 90% confidence intervals (CIs) were calculated for AUC_0-8h_, AUC_inf_, AUC_0-4min_, AUC_0-10min_, AUC_10-30min_ and C_max_. The treatment differences in Tmax were evaluated by a nonparametric approach (Wilcoxon signed rank test) on untransformed values. Power analysis was used to compare the bioavailability.

#### Sample Size

In-house data indicated a coefficient of variation (CV) for unconjugated naloxone Cmax of approximately 25%. Assuming a 26% intra-subject variability and a difference between the treatment means of 5% or less, the necessary sample size for a 90% probability of the 90% confidence interval of the treatment means ratio to be within the 80.00 to 125.00% range is estimated to be 40 subjects. Six (6) extra subjects were included into the study to account for potential dropouts, therefore, 46 subjects were enrolled and 42 completed the study and were included in the statistical analyses.

## Results

### FMXIN001 New Dry Powder-Based Formulation Characterization

**Fig. 1:**
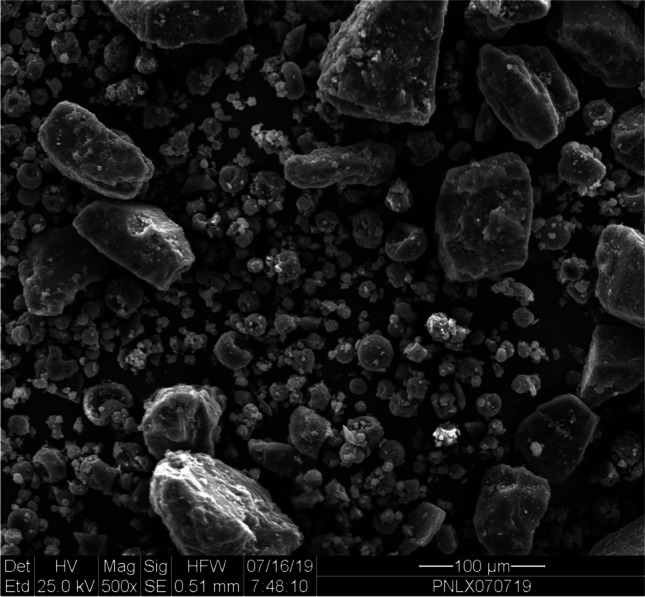
SEM image of FMXIN001 new dry powder-based formulation

Scanning electron microscopy (SEM) analysis was performed on FMXIN001 and a representative image is shown in Fig. 1 in which the small spherical particles of naloxone hydrochloride having the narrow size distribution of 5–30 μm are dispersed between large irregular particles of lactose ranging between 40 μm to 240 μm.

**Fig. 2:**
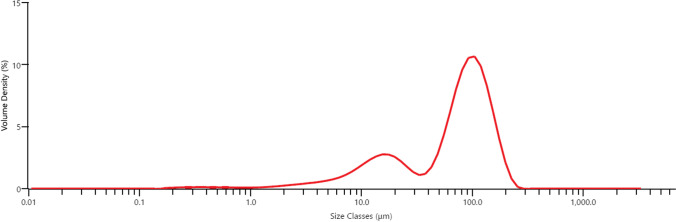
Particle size distribution FMXIN001 new dry powder-based formulation

The FMXIN001 formulation was subjected to particle size analysis using the Malvern Laser Diffraction instrument. The particle size distribution is shown in (Fig. 2), with the following density volumes (%) obtained for the particle sizes indicated: D (10) = 10.5 μm, D (50) = 77.7 μm and D (90) = 144 μm. The amount of particles obtained, less than 10 μm in size, was about 9.5% v/v and of less than 5 μm in size was about 4.9% v/v.

### Dose Uniformity

Ten devices were packed, activated, and the powder delivered upon actuation of each device (shot weight) was collected and weighed. Based on weights (mg) of the delivered powder dose from ten devices the uniformity of the delivered dose (mg/device and %) from each of the ten devices, was measured by HPLC. The average shot weight was 19.51 mg ± 2.49%, the average Naloxone content/device was 3.78 mg ± 2.20%, and the average Naloxone content %/device was 96.92± 2.13%. The results are in accordance with USP <601> requirements.

### FMXIN001 Stability under Accelerated Aging Conditions

Stability of FMXIN001 after loading into the Aptar device was determined after storage for 6 months at 40°C ± 2°C and 75% ±5% Relative Humidity (RH). All results were within specifications and total impurities after 6 months reached 0.8%.

### Phase Analysis by X-Ray Powder Diffraction

X-ray diffraction (XRD) pattern images of naloxone HCl raw material, lactose monohydrate or naloxone microspheres mixed with lactose monohydrate (batch PNLX070729) are shown in the on-line resource Fig. S1. Naloxone HCl peaks are not observed in the XRD pattern for naloxone microspheres, with the peaks determined due to lactose monohydrate, consistent with an amorphous microsphere structure. Following storage for 6 months, the amorphous microsphere structure is maintained, as judged by XRD, shown in on-line resource Fig. S2.

### Dry-Powder Deposition *In Vitro* Nasal Cast Study

**Fig. 3:**
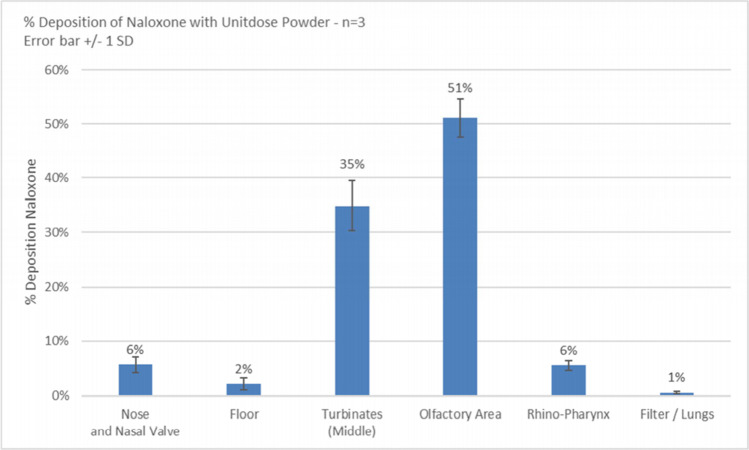
Mean of percentage deposition of naloxone microspheres dry powder formulation in each nasal cast, by region. Nasal cast used courtesy of Aptar Pharma, DTF medical and University of Tours

Results for the distribution of the naloxone dry powder formulation in a nasal cast following actuation of the Aptar Unit Dose powder device into two nostrils is shown in Fig. 3, with permission from Aptar Pharma, France. Eighty-six percent (86%) of the administered quantity of naloxone reached the turbinates region with 35% in the middle part and 51% in the upper part (olfactory area). There was no deposition in the lungs (less than 1%) and less than 10% in the nasopharynx.

### Pilot Clinical Study Results

Results from the preliminary pilot study in 14 healthy adult subjects (on-line resource Table S.II), summarized briefly here, strengthened Nasus Pharma’s working assumption that the newly developed drug delivery system is either comparable or superior to the reference product while maintaining the effectiveness of the treatment.

Overall, FMXIN001 displayed greater peak and total systemic exposure, with earlier onset of action (supported by greater partial exposures) for total and unconjugated naloxone when compared to the reference product, Narcan® (naloxone HCl) nasal spray 4 mg (Adapt Pharma, Inc., USA), after a single dose in healthy subjects under fasted conditions.

The administration of the study drug was generally well-tolerated by the healthy subjects who participated in this study. Overall, 9 mild TEAEs affecting 4 subjects (28.6% of subjects dosed) were reported during the conduct of the study (21.4% following treatment A and 21.4% following treatment B), all of which had a possible relationship to the investigational medicinal product (IMP), and were not related to the study device. No local irritation in the nasal cavity was observed or reported by the subjects. No SAEs were reported during the conduct of this study and none of the AEs had a significant impact on the safety of the subjects or on the integrity of the study results.

### Pivotal Study Demographics

Healthy male and female adult subjects were recruited into the pivotal study; the population demographics are described in Table I.

**Table II:** Summary of Study Results Based on Plasma Unconjugated Naloxone Levels

Variable	Trt	n	Arithmetic Mean (CV%)	Geometric Mean	Contrast	Ratio (%)	90% Confidence Interval	Intra-Sbj CV(%)	Power (%)
AUC_t_	A	42	13.6021 (33)	13.0188	A vs B	100.32	94.38–106.63	17	>99.99
(hr*ng/mL)	B	42	13.7503 (38)	12.9777					
AUC_inf_	A	42	13.8286 (33)	13.2355	A vs B	100.35	94.49–106.57	16	>99.99
(hr*ng/mL)	B	42	13.9849 (38)	13.1897					
C_max_	A	42	10.1262 (42)	9.3761	A vs B	113.01	102.73–124.32	26	53.79
*(ng/mL)*	B	42	8.7238 (32)	8.2965					
AUC_0-4min_	A	42	0.1824 (88)	0.1159	A vs B	163.22	122.98–216.62	90	00.61
(hr*ng/mL)	B	42	0.1207 (86)	0.0710					
AUC_0-10min_	A	42	0.9159 (67)	0.7306	A vs B	125.34	104.04–151.02	54	03.63
(hr*ng/mL)	B	42	0.7184 (57)	0.5828					
AUC_10-30min_	A	42	2.6958 (37)	2.5344	A vs B	113.52	104.74–123.04	22	62.86
(hr*ng/mL)	B	42	2.3211 (27)	2.2325					
Treatment A (Test)	Naloxone Intranasal Spray 4 mg (FMXIN001 4 mg microspheres powder, nasal spray), Lot No.: BPR-20-0013 (Nasus Pharma, Israel)								
Treatment B (Reference)	Narcan® (naloxone HCl) NASAL SPRAY 4 mg, Lot No.: 201804 (Adapt Pharma, Inc., USA)								

**Fig. 4:**
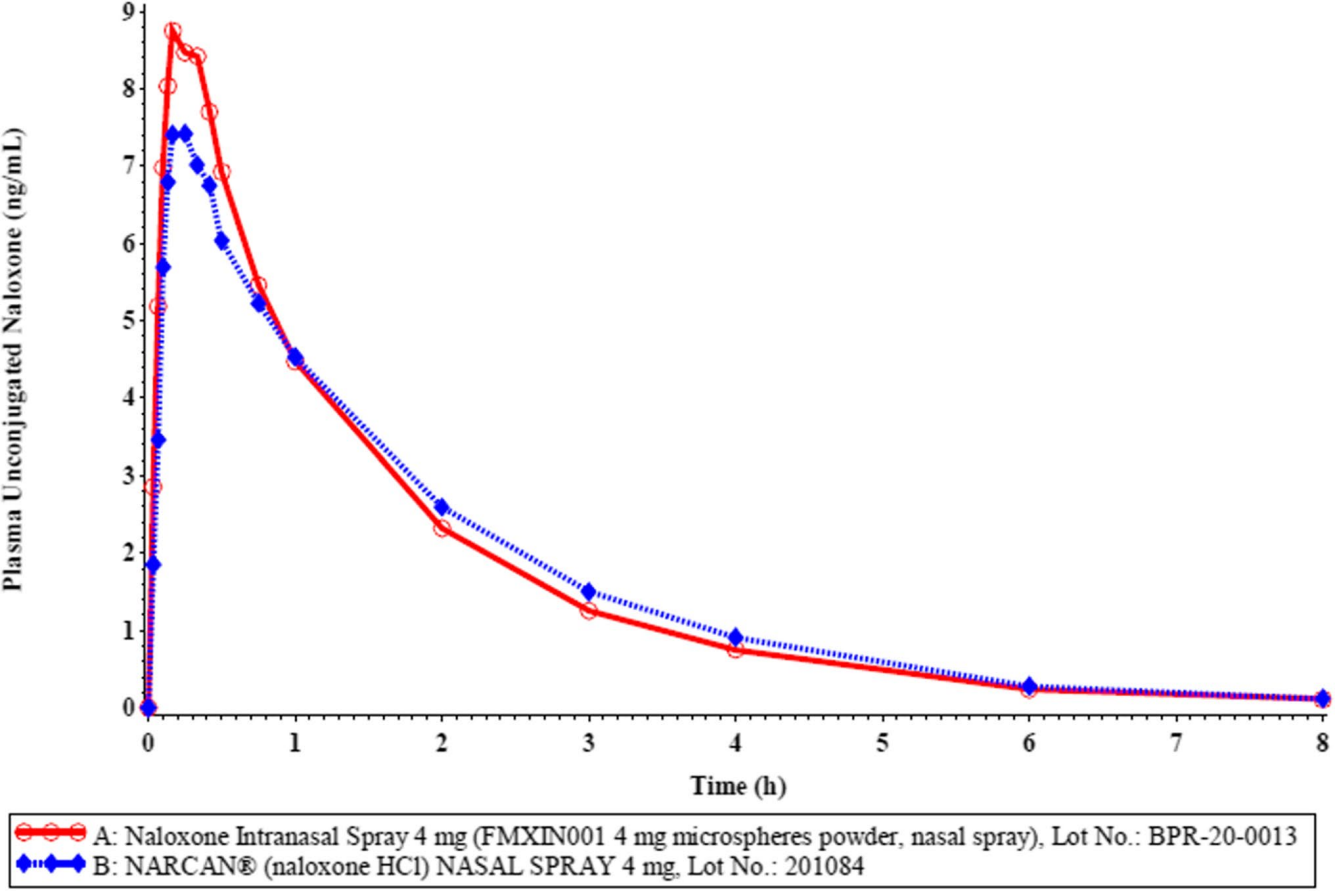
Mean Plasma Unconjugated Naloxone Concentration-Time Profile in a Linear Scale (A: n = 42 / B: n = 42)

**Fig. 5:**
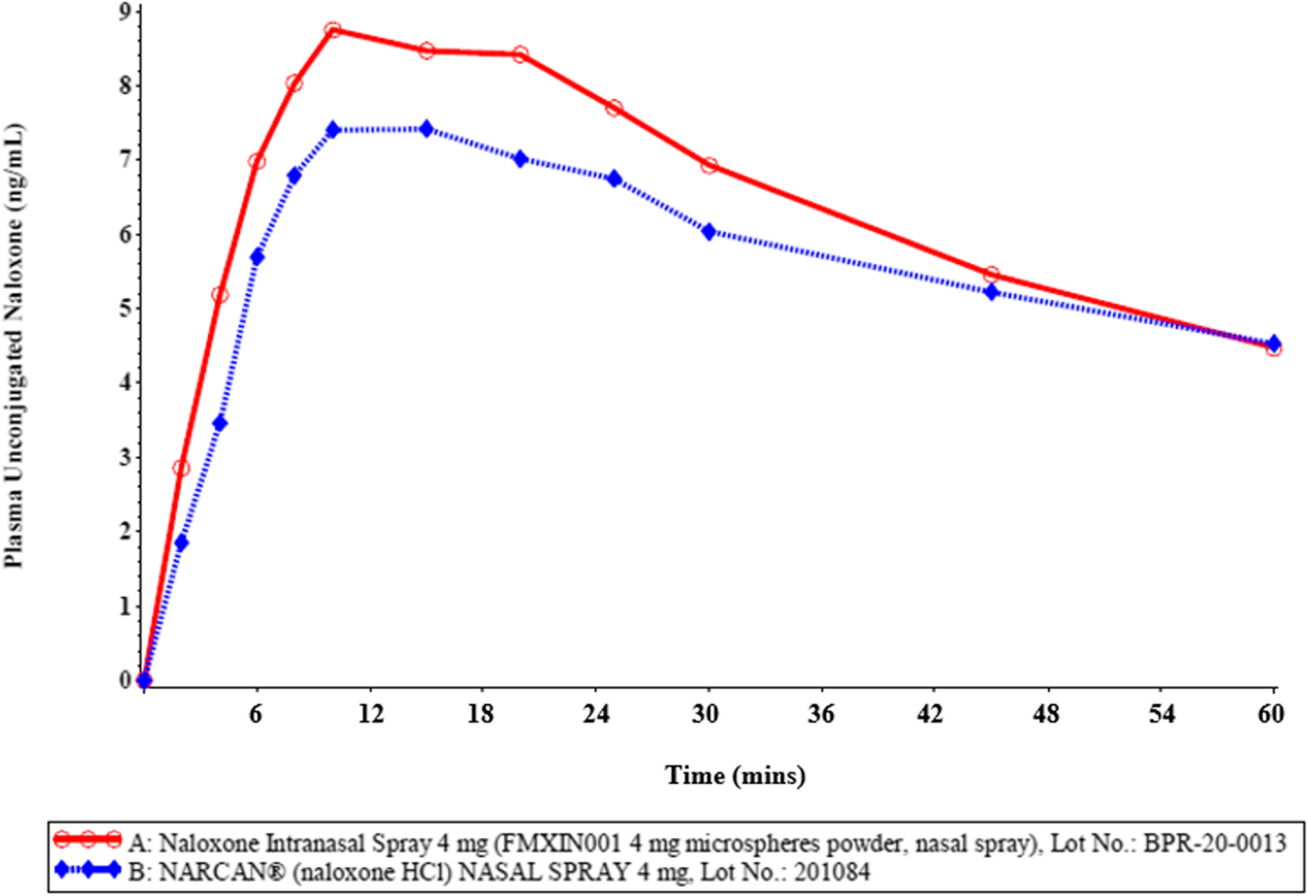
Mean Plasma Unconjugated Naloxone Concentration-Time Profile in a Linear Scale (A: n = 42 / B: n = 42) – First 1-Hour

AUCs determined over the whole time period or after extrapolation to infinity are very similar for FMXIN001 compared to Narcan®. There is no statistical difference between treatments A and B in AUC_t_ or AUC_inf_ (power > 99.99%) (Table II). As shown in Table II the AUCs calculated for the very early time points up to 4, 10 and 30 mins are greater for FMXIN001 compared to Narcan®, also evident in Figs. 4 and 5. Cmax is not equal, treatment A has higher Cmax (reflected in lower power 53.79%). The AUC 0–4 min and AUC 0-10 min, are not equal, (very low power 0.61%, 03.63%), clearly indicating lack of bioequivalence at the early time points. The nonparametric analysis of unconjugated naloxone T_max_ is presented in Table III. The Tmax does not differ significantly (Table III).

**Table III:** Nonparametric Analysis of Unconjugated Naloxone Tmax/ Contrast: A vs. B

Variable	Median A	Median B	Signed Rank S	P value ^*^
T_max_ (hr)	0.25	0.21	18	0.7631

^*^Based on Wilcoxon signed-rank test comparing distribution of T_max_ for Treatment A vs. distribution of T_max_ for Treatment B

Treatment A (Test): Naloxone Intranasal Spray 4 mg (FMXIN001 4 mg microspheres powder, nasal spray), Lot No.: BPR-20-0013 (Nasus Pharma, Israel).

Treatment B (Reference): Narcan® (naloxone HCl) NASAL SPRAY 4 mg, Lot No.: 201804 (Adapt Pharma, Inc., USA).

### Pivotal Study – Pharmacokinetic Effect and Statistical Analysis

The mean plasma unconjugated naloxone concentration-time profile is shown in Fig. 4 and the profile for the first hour is expanded in Fig. 5. Additional graphs with standard deviation, and at log scale are found in the on-line resource (Figs. S4 -S7).

Descriptive statistics from the pivotal study of naloxone treatments A and B for plasma unconjugated naloxone PK variables are summarized in Table II and further detailed in the on-line resource Table S.III.

#### Conclusions from PK Statistical Analysis

A significant treatment effect was detected by ANOVA in the analysis of C_max_ (p = 0.0368), AUC_0-4min_ (p = 0.0058), AUC_0-10min_ (p = 0.0478), and AUC_10-30min_ (p = 0.0113).

ANOVA did not detect a significant difference in any of the unconjugated naloxone PK variables for period or sequence effects, indicating that the order of test item or reference product administration, does not impact on the results.

### Pivotal Study Safety

Generally, the treatment and reference product were well tolerated with no significant adverse events and few mild self-resolving side effects, with similar frequency between treatment and reference groups; 41.3% occurred following administration of treatment A, and 37.0% occurred following administration of treatment B (see on-line resource Tables S.IV, S.V, S.VI and S.VII). All TEAEs were mild in severity and resolved prior to end-of-study without intervention. Most mild AEs were related to nasal mucosal congestion and there were no safety concerns with regard to smell tests.

## Discussion

The burgeoning opioid crisis has prompted exploration of different approaches to effectively tackle the problem of opioid overdose in the community setting. The liquid spray Narcan®, amongst a number of other intranasal products has achieved some measure of success [22]. In addition to performance or efficacy, the product’s ease of use, handling and storage conditions are critical attributes for community-use formulations of naloxone, which are likely to be used by laypersons in emergency situations.

### Microspheres Dry Powder Naloxone IN Formulation

In an emergency situation, the non-invasive intranasal route is best suited for rapid absorption and ease of use. The inherent advantages of powder nasal drug delivery over solution based nasal product have been extensively researched and described in the literature [1]. The powder technology described here confers improved PK characteristics in terms of more rapid absorption. For naloxone, immediate delivery of higher doses is imperative due to the increased use of more potent synthetic opioids. Our FMXIN001 formulation addresses all these issues.

We report an improvement in nasal delivery of naloxone aimed at treating the medical emergency of opioid overdose, over the liquid nasal spray approach by substituting a novel microspheres dry powder formulation, for the approved liquid naloxone formulation. Advantages include a significantly higher naloxone blood exposure immediately after administration due to the dry powder formulation’s distribution to the upper anterior layers of the nasal cavity, in particular to the upper cavity layers beyond the nasal valve. It has been suggested that liquid nasal sprays may have suboptimal absorption because a large fraction of the delivered drug is deposited in the anterior segment of the nasal cavity in front of the nasal valve [11]. This anterior segment is predominantly lined with non-ciliated squamous epithelium that is less permeable to drugs than the respiratory mucosa beyond the nasal valve [10].

It has also been suggested that nasal powders may improve drugs’ therapeutic effects by providing higher resistance against the mucociliary clearance, that may prolong the contact time for drug absorption [2, 23, 24]. Powder-based IN formulations apparently reach the blood stream more quickly and have better bioavailability than liquid sprays due to significantly larger deposition in the nasal mucosa [9].

### Particle Engineering

Particle engineering with lactose excipient is well known in the field of dry powder inhalation to the lungs (see for example, [25, 26]). Its application specifically for intranasal delivery is, however, less common. The FMXIN001 unique Microspheres Powder formulation is comprised of uniform microspheres (10-30 μm) together with much larger lactose carrier particles (Fig. 1) which enables deposition beyond the nasal valve, but the particle size precludes distribution into the airways or lungs. Upon intranasal delivery with the Aptar device the smaller naloxone microspheres may collide with the larger lactose particles to increase their distribution to the upper mucosa, as was found by *in vitro* deposition experiment using a nasal cast (Fig. 3). As well as serving as a carrier, the lactose serves as a disaggregating agent which may aid in maintaining the amorphous nature of naloxone microspheres, likely contributing to the product’s physical stability, expressed in particle size distribution (PSD), delivered doses uniformity and aerodynamic PSD. Disaggregation is important to keep the powder flowability and the carrier capacity of the lactose is helpful in assuring the full dosage of the powder is sprayed from the device.

In addition, the lactose carrier in the formulation may promote mucous secretion which aids dissolution of the powder and fast absorption of the drug [24]. Results from the *in vitro* deposition study (Fig. 3) indicate that, indeed, the microspheres powder formulation accumulates mainly in the middle turbinates region (35%) and olfactory area in the upper part of the nasal cavity (51%) of the cast.

These data contrast with published liquid spray distribution studies, in which a significant portion of the drug is deposited on the cavity floor before the nasal valve [11]. Recently, the value of nasal cast *in vitro* deposition studies has been brought into question [16, 23], with the authors recommending *in vivo* assessment when a definitive determination of nasal delivery performance is required. The dimensions of the nasal cavity increase with age and are, on average, larger in adult males compared to females. Additionally, changes in nasal deposition efficiencies can be influenced by the ethnicity of the subject [27].

Nevertheless, our clinical studies included both male and female volunteers from multiple ethnic groups, all of them received both the powder and the compared reference liquid formulations. This method reduces the impact of the gender, age, and ethnic differences in the nasal cavity and endorse the findings.

### Clinical Studies

Pilot and pivotal open-label, single-dose, randomized, two-period, two-treatment, two-sequence, crossover studies, evaluated the bioavailability profile of FMXIN001 compared to Narcan® reference product in a total of 56 (14 + 42) healthy adult subjects. The pivotal study population was heterogenous with a reasonable distribution across all variables, but with slight bias in favor of more female than male subjects (Table I), which was not considered to impact the study conclusions.

### Bioavailability of Dry Powder and Liquid Naloxone Intranasal Spray

Our results indicate with 90% confidence that, in general, the relative mean plasma unconjugated naloxone C_max_, AUC_0-8h_, AUC_inf_, and AUC_10-30min_ for treatment A (FMXIN001) were all within 80–125% of treatment B (Narcan®), which served as a reference. Furthermore, the pharmacokinetics of Treatment B (Narcan®) determined here, are consistent with those reported (Narcan® prescribing information), however, the PK profile in the first half hour after dosing was significantly better for FMXIN001 indicating a quicker and more relevant dosing effect in an emergency situation with short therapeutic window. Levels of naloxone obtained with FMXIN001 are consistent with therapeutic naloxone levels (Narcan® prescribing information).

### Pharmacokinetics – Faster Nasal Absorption of Dry Powder Formulation Vs. Liquid

Significantly greater partial exposure (AUC) is evident from our PK data for very early time points: 0–4 min, 0–10 min and 10–30 min, although the Tmax values were not significantly different. In the presented studies healthy volunteers were not exposed to opioids due to ethical reasons and hence pharmacodynamics of naloxone was not determined. Nevertheless, more rapid naloxone onset, as evident by the early higher exposure, could improve overdose outcomes in an emergency real-world situation, when time is of the essence.

The fundamental properties of a powder, its particle size and shape determine its flowability and dissolution. The uniform spherical shape of our Naloxone powder formulation and its excipient determine the therapeutic efficacy by influencing the absorption area in the nose and the rate of dissolution of the Naloxone and hence the therapeutic outcome.

Studies of Opioid overdose showed consistent need for higher doses and the need to repeat administration within the first few minutes after initial dosing of Naloxone, especially in view of the newer synthetic extra strong opioids. About a third of overdose victims needed a second dose and about 19% needed a third dose [12]. Biopharmaceutically, this means that higher absorbed doses of Naloxone, especially in the immediate minutes of therapy, are beneficial in restoring respiration and help resuscitate the patients.

### Safety

The naloxone administration in our pivotal study was generally well-tolerated by the healthy subjects. No serious adverse events (SAEs) were reported and none of the AEs had a significant impact on the safety of the subjects or on the integrity of the study results. No effect of the treatment was recorded on the sense of smell by validated smell test [18, 19].

It is reported that two or even three doses are sometimes required to treat overdose patients [12]. In such cases, the new powder formulation is not expected to affect the nasal mucosa since lactose safety as a nasal excipient had been established before in large controlled clinical studies. For example, lactose powder was used as the placebo formulation in the pivotal Phase 3 (TARGET) study of powder nasal sumatriptan that led eventually to its approval by the US FDA [28]. The TARGET study was a pivotal study that evaluated the safety and efficacy of nasal powder sumatriptan in a device vs a similar device loaded with lactose (22 mg). Two hundred and thirty patients (116 AVP-825 and 114 placebo device) participated in the study. The dosage was one time inhalation when the patient suffered a migraine attack. The most commonly reported adverse events (AEs) (≥ 2% in any treatment group) were abnormal product taste (22% AVP-825 vs 4% placebo device), nasal discomfort (13% vs 2%), rhinorrhea (5% vs 3%), and rhinitis (3% vs 0%).

In another clinical study lactose was used as a carrier for nasal inhaled fluocortin butyl drug in perennial allergic rhinitis in 306 patients (170 females and 136 males) in a large multi-center double blind placebo-controlled study. The patients were randomized into placebo – (lactose) inhalers (63 patients) and 3 treatment groups that received the drug (2, 4 and 8 mg). The treatment group assigned to receive 8 mg was further divided to two formulations containing the same amount of the active molecule but with different amounts of lactose. The different dosages of lactose were to determine if different amounts of this carrier affected the drug delivery. All in all, the patients in this last group were exposed to extremely high dosages of lactose (up to 336 mg/day) and no adverse events were recorded [9].

## Conclusion

The new stable and safe naloxone microspheres powdered formulation (FMXIN001) described here provides a significant and clinically meaningful advance beyond the currently marketed intranasal spray formulation, Narcan®. In terms of PK profile, the dry powder-based IN naloxone spray (FMXIN001) is comparable or superior to the reference liquid spray product (Narcan®), and is equally safe. The useability of the powder device was equal to the liquid device. The more rapid systemic appearance of unconjugated naloxone administered as the dry powder formulation represents a major step forward towards facilitating the rapid emergency treatment of opioid overdose subjects in the community environment. The clinical utility of the lactose-based dry powder intranasal delivery system, described here for naloxone, likely may be applicable to other agents in which exceedingly rapid bioavailability coupled with ease of use and stability are prerequisites.

## Supplementary Information

ESM 1 (DOCX 202 kb)

ESM 2 (DOCX 283 kb)

## Abbreviations

ADE: Adverse device event
AUC: Area under the curve
CI: Confidence interval
IM: Intramuscular
IMP: Investigational medicinal product
IN: Intranasal
IV: Intravenous
SC: Subcutaneous
PK: Pharmacokinetics
PSD: Particle size distribution
TEAE: Treatment-related adverse event
