# Supplementary material for: A Novel Faster-Acting, Dry Powder-Based, Naloxone Intranasal Formulation for Opioid Overdose
Source: Pharm Res. 2022 Apr 6;39(5):963–75. doi: 10.1007/s11095-022-03247-5 (PMC9160115; doi:10.1007/s11095-022-03247-5)
Supplement: Supplementary file 1 — (DOCX 202 kb) [file 11095_2022_3247_MOESM1_ESM.docx]

**Table S.I: Validation of the Stability-Indicating HPLC Methods for the Analysis of Naloxone IN Product**

| **Parameter** | **Naloxone (for assay)** | **Noroxymorphone** | **3-O-Allylnaloxone** | **10α-Hydroxynaloxone** | **2,2-Bisnaloxone** |
| --- | --- | --- | --- | --- | --- |
| Precision  (% RSD)* | 2 | 2 | 1 | 2 | 1 |
| Accuracy  (% recovery)* | 100 | 97 | 102 | 105 | 101 |
| LOQ (μg/mL) | - | 0.339 | 0.391 | 0.356 | 0.370 |
| LOD (μg/mL) | - | 0.015 | 0.029 | 0.01 | 0.023 |
| Regression equation | | | | | |
| Slope | - | 20541.2 | 19599.3 | 24750.2 | 13256.3 |
| Intercept | - | 109.6 | -171.2 | -84.6 | -362.7 |
| r^2^ value | - | 0.9998 | 0.9997 | 0.9999 | 0.9998 |

* Using 100% level sample (40 μg/mL) for assay and LOQ for related substances

**Table S.II: Pilot Study Summary of Study Results Based on Plasma Unconjugated Naloxone Levels**


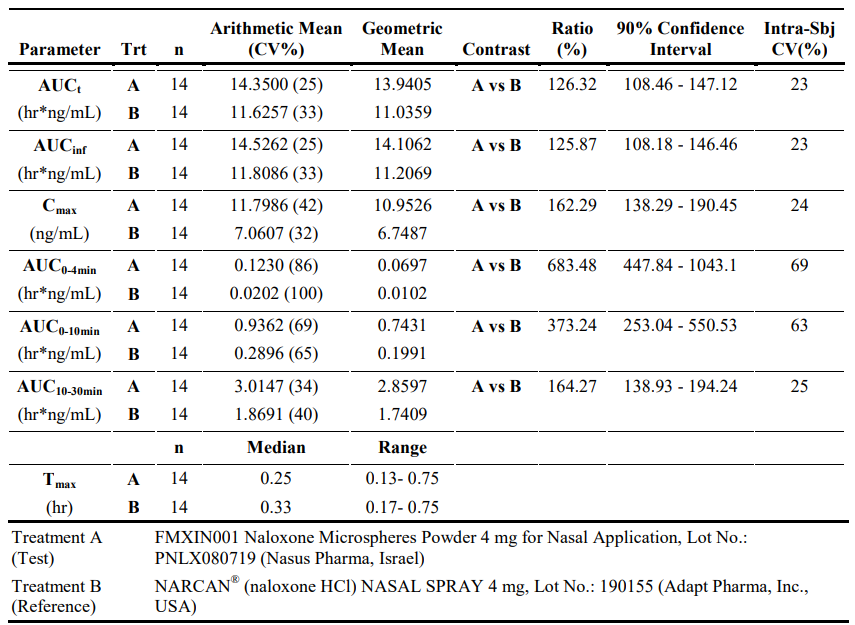


AUC_t_ refers to AUC_0-8hr_

**Table S.III: Pivotal Study Descriptive Statistics for Plasma Unconjugated Naloxone Pharmacokinetic Parameters**

| ***Parameter*** | ***Trt*** | ***GeoMean*** | ***ArithMean*** | ***SD*** | ***CV%*** | ***Median*** | ***Minimum*** | ***Maximum*** | ***N*** |
| --- | --- | --- | --- | --- | --- | --- | --- | --- | --- |
| **AUC_0-4min_** | **A** | 0.1167 | 0.1824 | 0.1598 | 87.61 | 0.1304 | 0.0140 | 0.6623 | 42 |
| *(hr*ng/mL)* | **B** | 0.0712 | 0.1207 | 0.1039 | 86.13 | 0.1002 | 0.0057 | 0.3618 | 42 |
| **AUC_0-10min_** | **A** | 0.7344 | 0.9159 | 0.6093 | 66.52 | 0.8098 | 0.1782 | 2.8786 | 42 |
| *(hr*ng/mL)* | **B** | 0.5843 | 0.7184 | 0.4112 | 57.24 | 0.7173 | 0.1334 | 1.6322 | 42 |
| **AUC_10-30min_** | **A** | 2.5331 | 2.6958 | 0.9855 | 36.56 | 2.4998 | 1.0299 | 5.5928 | 42 |
| *(hr*ng/mL)* | **B** | 2.2329 | 2.3211 | 0.6329 | 27.27 | 2.3370 | 1.0618 | 4.0164 | 42 |
| **AUC_t_** | **A** | 13.0145 | 13.6021 | 4.4402 | 32.64 | 12.6598 | 6.6632 | 31.5046 | 42 |
| *(hr*ng/mL)* | **B** | 12.9356 | 13.7503 | 5.2101 | 37.89 | 12.1199 | 6.1563 | 31.9715 | 42 |
| **AUC_inf_** | **A** | 13.2308 | 13.8286 | 4.5159 | 32.66 | 12.7833 | 6.7622 | 32.0422 | 42 |
| *(hr*ng/mL)* | **B** | 13.1469 | 13.9849 | 5.3324 | 38.13 | 12.2933 | 6.2123 | 32.5685 | 42 |
| **AUC_t_/AUC_inf_** | **A** | 98.37 | 98.37 | 1.18 | 1.20 | 98.60 | 94.16 | 99.59 | 42 |
| *(%)* | **B** | 98.39 | 98.39 | 0.57 | 0.58 | 98.52 | 97.21 | 99.37 | 42 |
| **C_max_** | **A** | 9.3795 | 10.1262 | 4.2816 | 42.28 | 9.5650 | 3.7500 | 26.6000 | 42 |
| *(ng/mL)* | **B** | 8.2965 | 8.7238 | 2.8276 | 32.41 | 8.3650 | 3.7100 | 17.7000 | 42 |
| **T_max_** | **A** | 0.23 | 0.26 | 0.13 | 49.53 | 0.25 | 0.10 | 0.75 | 42 |
| *(hr)* | **B** | 0.23 | 0.26 | 0.13 | 52.13 | 0.21 | 0.13 | 0.77 | 42 |
| **T_half_** | **A** | 1.32 | 1.34 | 0.24 | 17.67 | 1.32 | 0.96 | 2.06 | 42 |
| *(hr)* | **B** | 1.33 | 1.34 | 0.17 | 12.84 | 1.31 | 1.15 | 2.00 | 42 |
| **K_el_** | **A** | 0.5234 | 0.5305 | 0.0860 | 16.21 | 0.5262 | 0.3359 | 0.7247 | 42 |
| *(1/hr)* | **B** | 0.5205 | 0.5239 | 0.0582 | 11.12 | 0.5311 | 0.3460 | 0.6027 | 42 |
| **TLIN** | **A** | 1.51 | 1.86 | 1.08 | 58.06 | 2.00 | 0.25 | 4.00 | 42 |
| *(hr)* | **B** | 1.56 | 2.02 | 1.29 | 63.71 | 2.00 | 0.42 | 4.28 | 42 |
| **R²** | **A** | 0.9910 | 0.9911 | 0.0112 | 1.13 | 0.9944 | 0.9471 | 0.9998 | 42 |
|  | **B** | 0.9918 | 0.9919 | 0.0069 | 0.70 | 0.9935 | 0.9771 | 1.0000 | 42 |
| **LQCT** | **A** | 8.00 | 8.00 | 0.01 | 0.08 | 8.00 | 8.00 | 8.03 | 42 |
| *(hr)* | **B** | 8.00 | 8.00 | 0.02 | 0.19 | 8.00 | 8.00 | 8.08 | 42 |
| **C_t_** | **A** | 0.0942 | 0.1099 | 0.0684 | 62.26 | 0.0878 | 0.0321 | 0.3490 | 42 |
| *(ng/mL)* | **B** | 0.1032 | 0.1196 | 0.0709 | 59.28 | 0.0994 | 0.0337 | 0.3610 | 42 |
| *TLIN = start time for linear regression R² = coefficient of determination for regression analysis LQCT = time of the last quantifiable concentration C_t_ = last measurable concentration value at LQCT. This value was used for the extrapolation to infinity.*  *AUC_t_ = AUC_0-8hr_* **Treatment A: Naloxone Intranasal Spray 4 mg (FMXIN001 4 mg microspheres powder, nasal spray), Lot No.: BPR-20-0013 (Nasus Pharma, Israel) Treatment B: NARCAN^®^ (naloxone HCl) NASAL SPRAY 4 mg, Lot No.: 201804 (Adapt Pharma, Inc., USA)** | | | | | | | | | |

Table S.IV: Summary of All Treatment-Emergent Adverse Events for Each Treatment

|  | **Reported Incidence by Treatment Group** n (%) of Subjects | | |  | **Reported Frequency by Treatment Group** no. (%) of TEAEs | | |
| --- | --- | --- | --- | --- | --- | --- | --- |
|  | **A**  N = 46 | **B**  N = 43 | **Total**  N = 46 |  | **A**  No. = 29 | **B**  No. = 28 | **Total**  No. = 57 |
| Subjects with TEAEs | 19 (41.3%) | 17 (39.5%) | 28 (60.9%) |  | N/A | N/A | N/A |
| Subjects with No TEAEs | 27 (58.7%) | 26 (60.5%) | 18 (39.1%) |  | N/A | N/A | N/A |
|  | | | | | | | |
| **Severity** |  |  |  |  |  |  |  |
| Mild | 19 (41.3%) | 17 (39.5%) | 28 (60.9%) |  | 29 (100%) | 28 (100%) | 57 (100%) |
| Moderate | 0 (0%) | 0 (0%) | 0 (0%) |  | 0 (0%) | 0 (0%) | 0 (0%) |
| Severe | 0 (0%) | 0 (0%) | 0 (0%) |  | 0 (0%) | 0 (0%) | 0 (0%) |
|  | | | | | | | |
| **Relationship to IMP** |  |  |  |  |  |  |  |
| Unrelated | 3 (6.5%) | 6 (14.0%) | 8 (17.4%) |  | 3 (10.3%) | 6 (21.4%) | 9 (15.8%) |
| Possibly Related | 16 (34.8%) | 12 (27.9%) | 23 (50.0%) |  | 20 (69.0%) | 16 (57.1%) | 36 (63.2%) |
| Probably Related | 5 (10.9%) | 3 (7.0%) | 8 (17.4%) |  | 6 (20.7%) | 6 (21.4%) | 12 (21.1%) |
|  | | | | | | | |
| **Relationship to Study Device** |  |  |  |  |  |  |  |
| Not Applicable | 8 (17.4%) | 10 (23.3%) | 16 (34.8%) |  | 13 (44.8%) | 12 (42.9%) | 25 (43.9%) |
| Not Related | 0 (0%) | 0 (0%) | 0 (0%) |  | 0 (0%) | 0 (0%) | 0 (0%) |
| Possibly Related | 13 (28.3%) | 10 (23.3%) | 18 (39.1%) |  | 16 (55.2%) | 15 (53.6%) | 31 (54.4%) |
| Probably Related | 0 (0%) | 1 (2.3%) | 1 (2.2%) |  | 0 (0%) | 1 (3.6%) | 1 (1.8%) |
|  | | | | | | | |
| IMP, investigational medicinal product; MedDRA, Medical Dictionary for Regulatory Activities; N, number of subjects dosed; n, number of subjects in respective categories; N/A, not applicable; No., number of adverse events; no., number of adverse events in respective categories; TEAE, treatment-emergent adverse event.  For the ‘Reported Incidence’: - Although a subject may have 2 or more clinical adverse events under the same treatment, the subject is counted only once within a category. - The same subject may appear in different categories and treatments.  Since severity of ADEs and severity of other AEs were the same, the severity information was merged.  Adverse event terms are classified according to MedDRA Version 23.1 Treatment A: Naloxone Intranasal Spray 4 mg (FMXIN001 4 mg microspheres powder, nasal spray), Lot No.: BPR-20-0013 (Nasus Pharma, Israel) Treatment B: NARCAN^®^ (naloxone HCl) NASAL SPRAY 4 mg, Lot No.: 201084 (Adapt Pharma, Inc., USA) | | | | | | | |

Table S.V: Summary of Treatment-Emergent Adverse Events by System Organ Class and Preferred Term for Each Treatment

|  | **Reported Incidence by Treatment Group**  n (%) | | |  | **Reported Frequency by Treatment Group**  no. (%) | | |
| --- | --- | --- | --- | --- | --- | --- | --- |
| **System Organ Class**  Preferred Term | **A** N = 46 | **B** N = 43 | **Total** N = 46 |  | **A**  No. = 29 | **B**  No. = 28 | **Total**  No. = 57 |
| **Subjects with TEAEs** | **19 (41.3%)** | **17 (39.5%)** | **28 (60.9%)** |  | **N/A** | **N/A** | **N/A** |
| Subjects with No TEAEs | 27 (58.7%) | 26 (60.5%) | 18 (39.1%) |  | N/A | N/A | N/A |
|  | | | | | | | |
| **Eye disorders** | **2 (4.3%)** | **1 (2.3%)** | **2 (4.3%)** |  | **2 (6.9%)** | **1 (3.6%)** | **3 (5.3%)** |
| Lacrimation increased | 2 (4.3%) | 1 (2.3%) | 2 (4.3%) |  | 2 (6.9%) | 1 (3.6%) | 3 (5.3%) |
|  | | | | | | | |
| **Gastrointestinal disorders** | **0 (0%)** | **1 (2.3%)** | **1 (2.2%)** |  | **0 (0%)** | **1 (3.6%)** | **1 (1.8%)** |
| Tooth loss | 0 (0%) | 1 (2.3%) | 1 (2.2%) |  | 0 (0%) | 1 (3.6%) | 1 (1.8%) |
|  | | | | | | | |
| **General disorders and administration site conditions** | **2 (4.3%)** | **3 (7.0%)** | **5 (10.9%)** |  | **2 (6.9%)** | **3 (10.7%)** | **5 (8.8%)** |
| Catheter site bruise | 1 (2.2%) | 0 (0%) | 1 (2.2%) |  | 1 (3.4%) | 0 (0%) | 1 (1.8%) |
| Catheter site pain | 0 (0%) | 1 (2.3%) | 1 (2.2%) |  | 0 (0%) | 1 (3.6%) | 1 (1.8%) |
| Catheter site related reaction | 0 (0%) | 2 (4.7%) | 2 (4.3%) |  | 0 (0%) | 2 (7.1%) | 2 (3.5%) |
| Fatigue | 1 (2.2%) | 0 (0%) | 1 (2.2%) |  | 1 (3.4%) | 0 (0%) | 1 (1.8%) |
|  | | | | | | | |
| **Infections and infestations** | **1 (2.2%)** | **0 (0%)** | **1 (2.2%)** |  | **1 (3.4%)** | **0 (0%)** | **1 (1.8%)** |
| COVID-19 | 1 (2.2%) | 0 (0%) | 1 (2.2%) |  | 1 (3.4%) | 0 (0%) | 1 (1.8%) |
|  | | | | | | | |
| **Investigations** | **1 (2.2%)** | **1 (2.3%)** | **2 (4.3%)** |  | **1 (3.4%)** | **1 (3.6%)** | **2 (3.5%)** |
| Blood bilirubin increased | 1 (2.2%) | 0 (0%) | 1 (2.2%) |  | 1 (3.4%) | 0 (0%) | 1 (1.8%) |
| Electrocardiogram T wave inversion | 0 (0%) | 1 (2.3%) | 1 (2.2%) |  | 0 (0%) | 1 (3.6%) | 1 (1.8%) |
|  | | | | | | | |
| **Nervous system disorders** | **4 (8.7%)** | **4 (9.3%)** | **6 (13.0%)** |  | **5 (17.2%)** | **6 (21.4%)** | **11 (19.3%)** |
| Dizziness | 1 (2.2%) | 3 (7.0%) | 4 (8.7%) |  | 1 (3.4%) | 4 (14.3%) | 5 (8.8%) |
| Dysgeusia | 1 (2.2%) | 0 (0%) | 1 (2.2%) |  | 1 (3.4%) | 0 (0%) | 1 (1.8%) |
| Headache | 1 (2.2%) | 0 (0%) | 1 (2.2%) |  | 1 (3.4%) | 0 (0%) | 1 (1.8%) |
| Hypoaesthesia | 0 (0%) | 1 (2.3%) | 1 (2.2%) |  | 0 (0%) | 1 (3.6%) | 1 (1.8%) |
| Paraesthesia | 1 (2.2%) | 0 (0%) | 1 (2.2%) |  | 1 (3.4%) | 0 (0%) | 1 (1.8%) |
| Presyncope | 1 (2.2%) | 1 (2.3%) | 2 (4.3%) |  | 1 (3.4%) | 1 (3.6%) | 2 (3.5%) |
|  | | | | | | | |
| **Respiratory, thoracic and mediastinal disorders** | **12 (26.1%)** | **9 (20.9%)** | **18 (39.1%)** |  | **18 (62.1%)** | **15 (53.6%)** | **33 (57.9%)** |
| Epistaxis | 0 (0%) | 1 (2.3%) | 1 (2.2%) |  | 0 (0%) | 2 (7.1%) | 2 (3.5%) |
| Nasal congestion | 3 (6.5%) | 2 (4.7%) | 5 (10.9%) |  | 4 (13.8%) | 3 (10.7%) | 7 (12.3%) |
| Nasal discomfort | 0 (0%) | 1 (2.3%) | 1 (2.2%) |  | 0 (0%) | 1 (3.6%) | 1 (1.8%) |
| Nasal inflammation | 0 (0%) | 1 (2.3%) | 1 (2.2%) |  | 0 (0%) | 1 (3.6%) | 1 (1.8%) |
| Nasal mucosal disorder | 11 (23.9%) | 6 (14.0%) | 14 (30.4%) |  | 11 (37.9%) | 6 (21.4%) | 17 (29.8%) |
| Nasal pruritus | 1 (2.2%) | 0 (0%) | 1 (2.2%) |  | 1 (3.4%) | 0 (0%) | 1 (1.8%) |
| Rhinalgia | 0 (0%) | 1 (2.3%) | 1 (2.2%) |  | 0 (0%) | 1 (3.6%) | 1 (1.8%) |
| Rhinorrhoea | 1 (2.2%) | 1 (2.3%) | 2 (4.3%) |  | 1 (3.4%) | 1 (3.6%) | 2 (3.5%) |
| Sneezing | 1 (2.2%) | 0 (0%) | 1 (2.2%) |  | 1 (3.4%) | 0 (0%) | 1 (1.8%) |
|  | | | | | | | |
| **Vascular disorders** | **0 (0%)** | **1 (2.3%)** | **1 (2.2%)** |  | **0 (0%)** | **1 (3.6%)** | **1 (1.8%)** |
| Hypertension | 0 (0%) | 1 (2.3%) | 1 (2.2%) |  | 0 (0%) | 1 (3.6%) | 1 (1.8%) |
|  | | | | | | | |
| MedDRA, Medical Dictionary for Regulatory Activities; N, number of subjects dosed; n, number of subjects in respective categories; N/A, not applicable; No., number of adverse events; no., number of adverse events in respective categories; PT, preferred term; SOC, system organ class; TEAE, treatment-emergent adverse event.  For the ‘Reported Incidence’:  - Subjects having 2 or more adverse events under the same treatment are counted only once within a category.  - The same subject may appear in different categories and treatments.  Adverse event terms are classified according to MedDRA Version 23.1 and sorted alphabetically by both SOC and PT.  Treatment A: Naloxone Intranasal Spray 4 mg (FMXIN001 4 mg microspheres powder, nasal spray), Lot No.: BPR-20-0013 (Nasus Pharma, Israel) Treatment B: NARCAN^®^ (naloxone HCl) NASAL SPRAY 4 mg, Lot No.: 201084 (Adapt Pharma, Inc., USA) | | | | | | | |

Table S.VI: Summary of Drug-Related Adverse Events for Each Treatment

|  | **Reported Incidence by Treatment Group** n (%) of Subjects | | |  | **Reported Frequency by Treatment Group** no. (%) of TEAEs | | |
| --- | --- | --- | --- | --- | --- | --- | --- |
|  | **A**  N = 46 | **B**  N = 43 | **Total**  N = 46 |  | **A**  No. = 26 | **B**  No. = 22 | **Total**  No. = 48 |
| Subjects with Drug-Related AEs | 17 (37.0%) | 13 (30.2%) | 24 (52.2%) |  | N/A | N/A | N/A |
| Subjects with No Drug-Related AEs | 29 (63.0%) | 30 (69.8%) | 22 (47.8%) |  | N/A | N/A | N/A |
|  | | | | | | | |
| **Severity** |  |  |  |  |  |  |  |
| Mild | 17 (37.0%) | 13 (30.2%) | 24 (52.2%) |  | 26 (100%) | 22 (100%) | 48 (100%) |
| Moderate | 0 (0%) | 0 (0%) | 0 (0%) |  | 0 (0%) | 0 (0%) | 0 (0%) |
| Severe | 0 (0%) | 0 (0%) | 0 (0%) |  | 0 (0%) | 0 (0%) | 0 (0%) |
|  | | | | | | | |
| MedDRA, Medical Dictionary for Regulatory Activities; N, number of subjects dosed; n, number of subjects in respective categories; N/A, not applicable; No., number of adverse events; no., number of adverse events in respective categories; TEAE, treatment-emergent adverse event.  Drug-related AEs include TEAEs which are possibly or probably related to the investigational medicinal product, whether or not the relationship was attributed to other source (eg, study device) simultaneously.  For the ‘Reported Incidence’: - Although a subject may have 2 or more clinical adverse events under the same treatment, the subject is counted only once within a category. - The same subject may appear in different categories and treatments.  Adverse event terms are classified according to MedDRA Version 23.1 Treatment A: Naloxone Intranasal Spray 4 mg (FMXIN001 4 mg microspheres powder, nasal spray), Lot No.: BPR-20-0013 (Nasus Pharma, Israel) Treatment B: NARCAN^®^ (naloxone HCl) NASAL SPRAY 4 mg, Lot No.: 201084 (Adapt Pharma, Inc., USA) | | | | | | | |

**Table S.VII: Summary of Drug-Related Adverse Events by System Organ Class and Preferred Term for Each Treatment**

|  | **Reported Incidence by Treatment Group**  n (%) | | |  | **Reported Frequency by Treatment Group**  no. (%) | | |
| --- | --- | --- | --- | --- | --- | --- | --- |
| **System Organ Class**  Preferred Term | **A** N = 46 | **B** N = 43 | **Total** N = 46 |  | **A**  No. = 26 | **B**  No. = 22 | **Total**  No. = 48 |
| **Subjects with Drug-Related AEs** | **17 (37.0%)** | **13 (30.2%)** | **24 (52.2%)** |  | **N/A** | **N/A** | **N/A** |
|  | | | | | | | |
| **Eye disorders** | **2 (4.3%)** | **1 (2.3%)** | **2 (4.3%)** |  | **2 (7.7%)** | **1 (4.5%)** | **3 (6.3%)** |
| Lacrimation increased | 2 (4.3%) | 1 (2.3%) | 2 (4.3%) |  | 2 (7.7%) | 1 (4.5%) | 3 (6.3%) |
|  | | | | | | | |
| **General disorders and administration site conditions** | **1 (2.2%)** | **0 (0%)** | **1 (2.2%)** |  | **1 (3.8%)** | **0 (0%)** | **1 (2.1%)** |
| Fatigue | 1 (2.2%) | 0 (0%) | 1 (2.2%) |  | 1 (3.8%) | 0 (0%) | 1 (2.1%) |
|  | | | | | | | |
| **Investigations** | **1 (2.2%)** | **1 (2.3%)** | **2 (4.3%)** |  | **1 (3.8%)** | **1 (4.5%)** | **2 (4.2%)** |
| Blood bilirubin increased | 1 (2.2%) | 0 (0%) | 1 (2.2%) |  | 1 (3.8%) | 0 (0%) | 1 (2.1%) |
| Electrocardiogram T wave inversion | 0 (0%) | 1 (2.3%) | 1 (2.2%) |  | 0 (0%) | 1 (4.5%) | 1 (2.1%) |
|  | | | | | | | |
| **Nervous system disorders** | **3 (6.5%)** | **3 (7.0%)** | **5 (10.9%)** |  | **4 (15.4%)** | **4 (18.2%)** | **8 (16.7%)** |
| Dizziness | 1 (2.2%) | 2 (4.7%) | 3 (6.5%) |  | 1 (3.8%) | 3 (13.6%) | 4 (8.3%) |
| Dysgeusia | 1 (2.2%) | 0 (0%) | 1 (2.2%) |  | 1 (3.8%) | 0 (0%) | 1 (2.1%) |
| Headache | 1 (2.2%) | 0 (0%) | 1 (2.2%) |  | 1 (3.8%) | 0 (0%) | 1 (2.1%) |
| Hypoaesthesia | 0 (0%) | 1 (2.3%) | 1 (2.2%) |  | 0 (0%) | 1 (4.5%) | 1 (2.1%) |
| Paraesthesia | 1 (2.2%) | 0 (0%) | 1 (2.2%) |  | 1 (3.8%) | 0 (0%) | 1 (2.1%) |
|  | | | | | | | |
| **Respiratory, thoracic and mediastinal disorders** | **12 (26.1%)** | **9 (20.9%)** | **18 (39.1%)** |  | **18 (69.2%)** | **15 (68.2%)** | **33 (68.8%)** |
| Epistaxis | 0 (0%) | 1 (2.3%) | 1 (2.2%) |  | 0 (0%) | 2 (9.1%) | 2 (4.2%) |
| Nasal congestion | 3 (6.5%) | 2 (4.7%) | 5 (10.9%) |  | 4 (15.4%) | 3 (13.6%) | 7 (14.6%) |
| Nasal discomfort | 0 (0%) | 1 (2.3%) | 1 (2.2%) |  | 0 (0%) | 1 (4.5%) | 1 (2.1%) |
| Nasal inflammation | 0 (0%) | 1 (2.3%) | 1 (2.2%) |  | 0 (0%) | 1 (4.5%) | 1 (2.1%) |
| Nasal mucosal disorder | 11 (23.9%) | 6 (14.0%) | 14 (30.4%) |  | 11 (42.3%) | 6 (27.3%) | 17 (35.4%) |
| Nasal pruritus | 1 (2.2%) | 0 (0%) | 1 (2.2%) |  | 1 (3.8%) | 0 (0%) | 1 (2.1%) |
| Rhinalgia | 0 (0%) | 1 (2.3%) | 1 (2.2%) |  | 0 (0%) | 1 (4.5%) | 1 (2.1%) |
| Rhinorrhoea | 1 (2.2%) | 1 (2.3%) | 2 (4.3%) |  | 1 (3.8%) | 1 (4.5%) | 2 (4.2%) |
| Sneezing | 1 (2.2%) | 0 (0%) | 1 (2.2%) |  | 1 (3.8%) | 0 (0%) | 1 (2.1%) |
|  | | | | | | | |
| **Vascular disorders** | **0 (0%)** | **1 (2.3%)** | **1 (2.2%)** |  | **0 (0%)** | **1 (4.5%)** | **1 (2.1%)** |
| Hypertension | 0 (0%) | 1 (2.3%) | 1 (2.2%) |  | 0 (0%) | 1 (4.5%) | 1 (2.1%) |
|  | | | | | | | |
| AE, adverse event; IMP, investigational medicinal product; MedDRA, Medical Dictionary for Regulatory Activities; N, number of subjects dosed; n, number of subjects in respective categories; N/A, not applicable; No., number of adverse events; no., number of adverse events in respective categories; PT, preferred term; SOC, system organ class.  Drug-related AEs include AEs with a possible or probable relationship to the IMP. For the ‘Reported Incidence’:  - Subjects having 2 or more adverse events under the same treatment are counted only once within a category.  - The same subject may appear in different categories and treatments.  Adverse event terms are classified according to MedDRA Version 23.1 and sorted alphabetically by both SOC and PT.  Treatment A: Naloxone Intranasal Spray 4 mg (FMXIN001 4 mg microspheres powder, nasal spray), Lot No.: BPR-20-0013 (Nasus Pharma, Israel) Treatment B: NARCAN^®^ (naloxone HCl) NASAL SPRAY 4 mg, Lot No.: 201084 (Adapt Pharma, Inc., USA) | | | | | | | |
