# Supplementary material for: A Novel Faster-Acting, Dry Powder-Based, Naloxone Intranasal Formulation for Opioid Overdose
Source: Pharm Res. 2022 Apr 6;39(5):963–75. doi: 10.1007/s11095-022-03247-5 (PMC9160115; doi:10.1007/s11095-022-03247-5)
Supplement: Supplementary file 2 — (DOCX 283 kb) [file 11095_2022_3247_MOESM2_ESM.docx]

**On-line resource: Supplementary Figures S1 -S7**

**Fig. S1 XRD pattern images for Lactose Monohydrate, Naloxone HCl and Naloxone HCl microparticles**


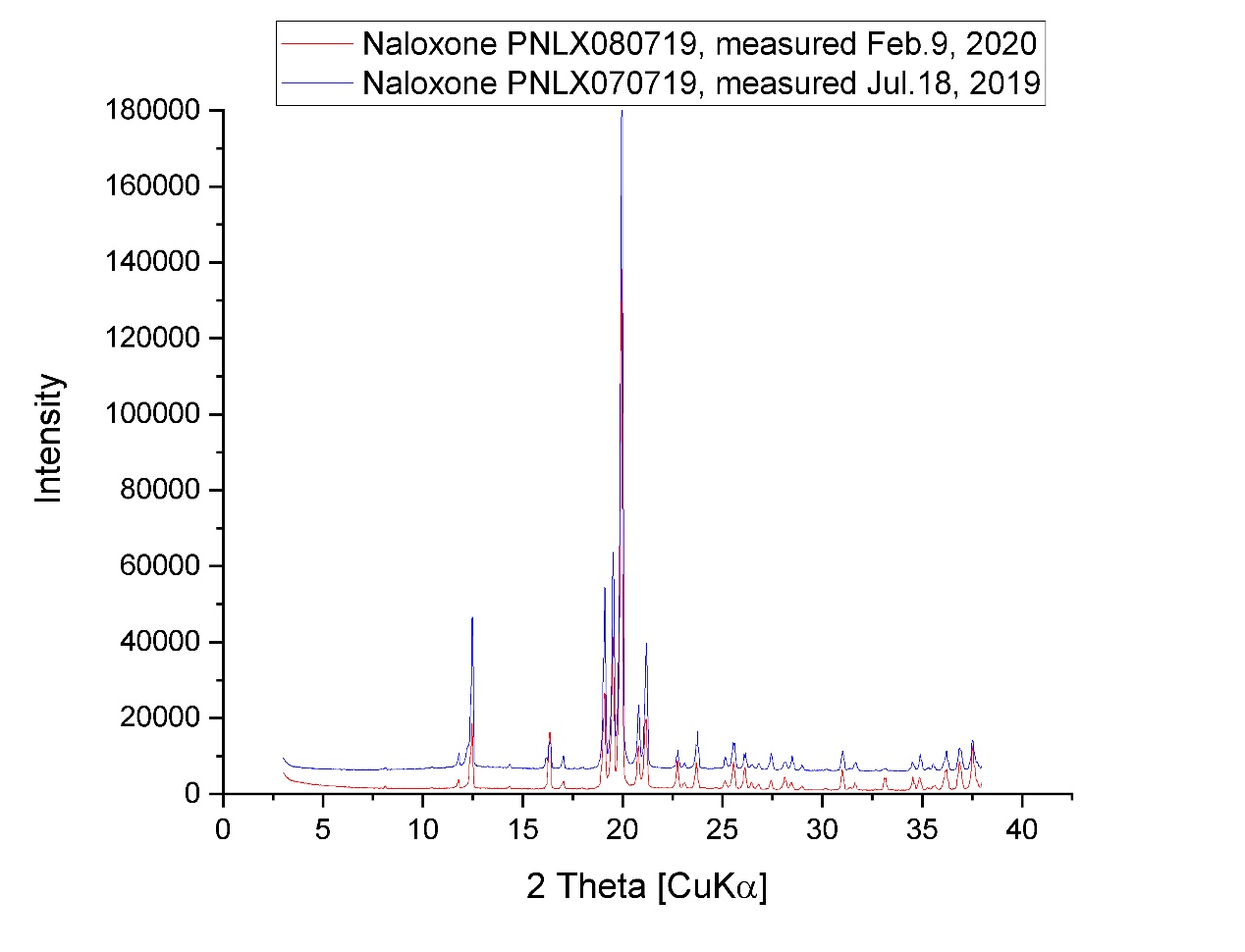


**Fig. S2 XRD pattern images for Initial Naloxone microsphere powder (lower) and stored for 6 months (upper)**


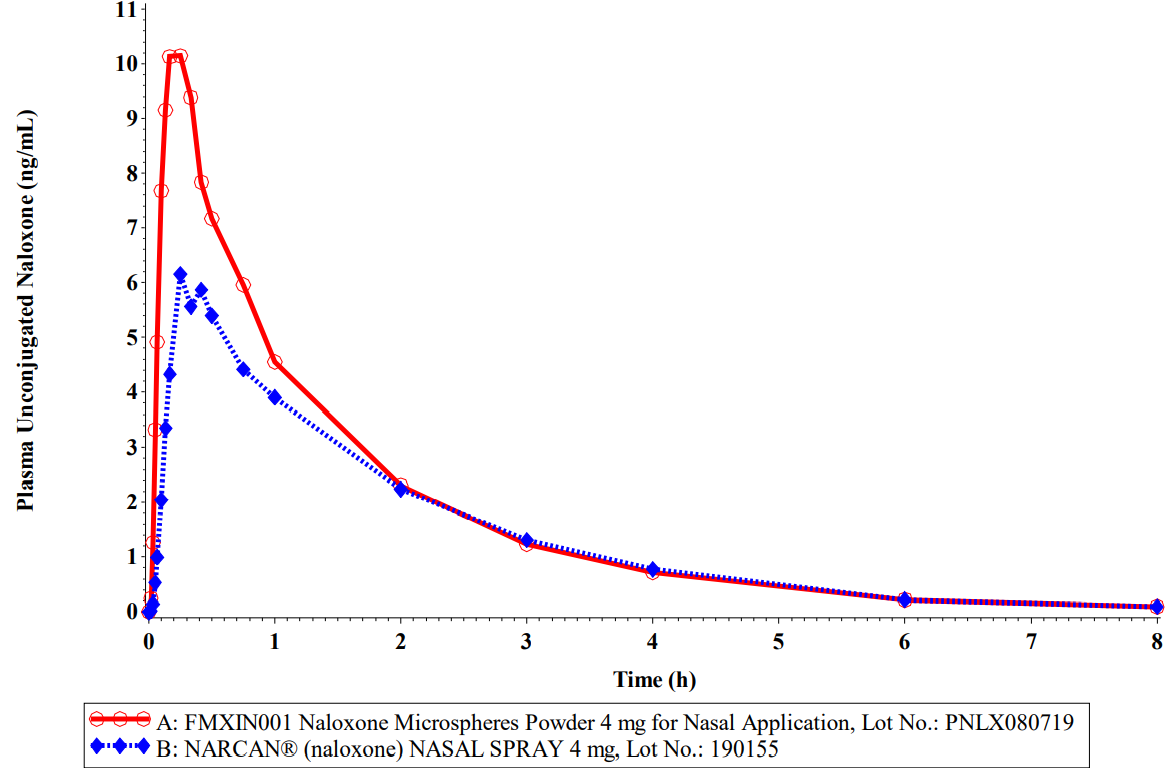


**Fig. S3 Pilot Study Mean Plasma Unconjugated Naloxone Concentration-Time Profile: Linear Scale (A: n = 14 / B: n = 14)**

**Fig. S4 Mean (±SD) Plasma Unconjugated Naloxone Concentration-Time Profile Linear Scale (A: n = 42 / B: n = 42)**

**Fig. S5 Mean (±SD) Plasma Unconjugated Naloxone Concentration-Time Profile Semi-Log Scale (A: n = 42 / B: n = 42)**

**Fig. S6: Mean (±SD) Plasma Unconjugated Naloxone Concentration-Time Profile Linear Scale (A: n = 42 / B: n = 42)**

**Fig. S7: Mean (±SD) Plasma Unconjugated Naloxone Concentration-Time Profile Semi-Log Scale (A: n = 42 / B: n = 42)**
